# Tranexamic acid and the reduction of blood loss in total knee and hip arthroplasty: a meta-analysis

**DOI:** 10.1186/1756-0500-6-184

**Published:** 2013-05-07

**Authors:** Rajiv Gandhi, Heather MK Evans, Safiyyah R Mahomed, Nizar N Mahomed

**Affiliations:** 1 Department of Orthopaedic Surgery, Toronto Western Hospital, 399 Bathurst St, 1-439 East Wing, Toronto, Ontario, Canada; 2 University of Toronto, Toronto Western Hospital, 399 Bathurst St, 1-439 East Wing, Toronto, Ontario, Canada; 3 MacMaster University, Toronto Western Hospital, 399 Bathurst St, 1-439 East Wing, Toronto, Ontario, Canada; 4 Department of Orthopaedic Surgery, Toronto Western Hospital, 399 Bathurst St, 1-439 East Wing, Toronto, Ontario, Canada

**Keywords:** Tranexamic acid, Antifibrinolytic, Allogeneic transfusions, Joint replacement

## Abstract

**Background:**

Tranexamic acid (TXA) is an antifibrinolytic drug used as a blood-sparing technique in many surgical specialties. The principal objective of our meta-analysis was to review randomized, controlled trials (RCT) comparing total blood loss and the number of patients receiving allogeneic blood transfusions with and without the use of TXA for knee (TKA) and hip (THA) arthroplasty.

**Methods:**

Studies were included if patients underwent primary unilateral TKA or THA; the study involved the comparison of a TXA treatment group to a control group who received either a placebo or no treatment at all; outcome measures included total blood loss TBL, number of patients receiving allogeneic blood transfusions, and/or incidence of thromboembolic complications; the study was a published or unpublished RCT from 1995 – July 2012.

**Results:**

Data were tested for publication bias and statistical heterogeneity. Combined weighted mean differences in blood loss favoured TXA over control for TKA and THA patients respectively [ −1.149 (*p* < 0.001; 95% CI −1.298, -1.000), -0.504 (*p <* 0.001; 95% CI, -0.672, -0.336)]. Combined odds ratios favoured fewer patients requiring allogeneic transfusions for TKA and THA with the use of TXA respectively [0.145 (*p <* 0.001; 95% CI, 0.094, 0.223), 0.327 (*p <* 0.001; 95% CI, 0.208, 0.515)]. Combined odds ratios indicated no increased incidence of DVT with TXA use in TKA and THA respectively [1.030 (*p* = 0.946; 95% CI, 0.439, 2.420), 1.070 (*p = 0.895*; 95% CI, 0.393, 2.911)].

**Conclusions:**

TXA should be considered for routine use in primary knee and hip arthroplasty to decrease blood loss.

## Background

The prevalence of total knee (TKA) and total hip arthroplasty (THA) is increasing and both are associated with considerable blood loss [1-9] thereby increasing a patient’s risk of transfusion [5,9,10]. Blood loss often leads to significant postoperative anemia [11] predisposing to an increased risk for cardiopulmonary events, transfusion reactions, and increased health care costs [5,12]. Allogeneic transfusions may also increase the patient’s risk for post-operative infection [3,10,13,14].

Tranexamic acid (TXA) is a synthetic amino acid [2-4,6,9,12,14-16] which competitively blocks the lysine binding sites on plasminogen and thereby slows the conversion of plasminogen to plasmin [2,3,5-8,10,12,14-20]. TXA may be administered intra-venous (IV) or topically in the surgical wound. TXA has been reported to reduce blood loss and be cost effective in many areas of orthopedic surgery, such as spinal surgery [11] as well as knee and hip arthroplasty [15,17-19]. One significant concern with TXA however, is the possibility that it, as well as other antifibrinolytics, could increase the risk of developing thromboembolic complications such as deep vein thrombosis (DVT).

We performed a meta-analysis of randomized, controlled trials (RCT) to assess the efficacy of TXA in TKA and THA for the outcomes of total blood loss (TBL), the number of patients receiving allogeneic transfusions and the incidence of DVT.

We hypothesized that the use of TXA in both TKA and THA would significantly reduce blood loss and the number of patients receiving allogeneic transfusions without an increased incidence of DVT.

## Methods

### Eligibility criteria

Studies were included in the meta-analysis if: 1) patients underwent primary unilateral TKA or THA; 2) the study involved the comparison of a TXA treatment group to a control group who received either a placebo or no treatment at all; 3) outcome measures included TBL, number of patients receiving allogeneic blood transfusions, and/or incidence of thromboembolic complications; 4) the study was a published or unpublished RCT from 1995 – July 2012; 5) the procedure involved was not described as ‘minimally invasive’ or ‘less invasive’. Both English and non-English studies were included in the meta-analysis. Data on cost-effectiveness was not included in many research papers and thus was not used as an eligibility criterion.

### Study identification

Two independent reviewers completed a systematic computerized search of online data-bases including Pubmed, Ovid MEDLINE and EMBASE. The key words used for the search included: *tranexamic acid* OR *TXA* AND *total knee replacement* OR *total hip replacement* OR *total knee arthroplasty* OR *total hip arthroplasty* OR *TKA* OR *THA* OR *TKR* OR *THR*. We also searched the Cochrane Database of Systematic Reviews, the Cochrane Central Register of Controlled Trials and http://www.Clinicaltrials.gov. After reviewing the title of the study we retrieved the abstract if we felt it was appropriate. We independently reviewed these abstracts and chose those studies that were potentially relevant. Bibliographies of each study included were reviewed for any further studies. Further, we searched the archives of the American Academy of Orthopedic Surgeons (2001–2011), Knee Society (2001–2011), Canadian Orthopedic Association (2003–2011), British Orthopedic Association (2002–2011) meetings for other potential studies.

### Assessment of study quality

All studies were reviewed by two independent reviewers using the Jadad Score, a scale of 0 (very poor) to 5 (rigorous) was used to assess the methodological strength of a clinical trial [21]. Any conflicts were resolved by consensus. Studies with a Jadad score of 1 were considered poor, scores of 2 were considered adequate and a score of 3 or higher was considered as high quality.

### Data collection

The following data were collected from all manuscripts for both the treatment group (TXA) as well as the control group: patient demographics, number of patients, dose of TXA, method of TXA administration, type of control (saline, non-TXA), TBL and/or number of patients receiving allogeneic transfusions, transfusion criteria, DVT screening method, thromboprophylaxis used, incidence of DVT and/or thromboembolic complications, as well as cost effectiveness.

### Statistical analysis

Knee and hip replacement data were analyzed separately for our outcomes of interest: total surgical blood loss and the number of patients requiring allogeneic blood transfusions. In both cases, data were aggregated using a random-effects model and the meta-analysis was performed using *Comprehensive Meta-analysis version 2.0 (Engelwood, NJ).*

The summary statistic used for the continuous outcome of mean blood loss was the weighted mean difference (WMD). The WMD refers to the ratio of the differences between the means of the treatment and control groups divided by the standard deviation. In our study, a negative WMD favored the treatment group (TXA) and a positive WMD favored the control group. Some studies did not provide standard deviation (SD) values so, whenever possible, these values were cited from a previous systematic review of antifibrinolytic therapies by Kagoma et al. [22] which used pooled and estimated SD values where studies had not provided them. In some cases, if a CI was provided, the SD was calculated using the following formula where U refers to the upper limit of the CI, μ to the mean, s to the SD and n to the number of study participants in each group (TXC, control).

$$\mathrm{U-\mu}=s/\mathrm{sqrt}\left(n\right)2$$

The summary statistic used for the outcomes: the number of patients requiring allogeneic transfusions as well as the incidence of DVT, was the odds ratio (OR). In this case, the OR referred to the odds of a patient requiring a blood transfusion in the treatment group divided by the odds of a patient requiring a blood transfusion in the control group; similarly, the odds of a patient having a DVT in the treatment group divided by the odds of a patient having a DVT in the control group. An OR value of less than one indicates that fewer patients in the TXA group required allogeneic blood transfusions, or developed a DVT, than in the control group. An OR value of greater than one indicates that more patients in the TXA group required allogeneic blood transfusions, or developed a DVT, than in the control group. We used two strategies to assess statistical heterogeneity between studies for both of our outcome measures. First, we used the methods of Hedges and Olkin to test for significance and homogeneity [23]. A p value < 0.1 was considered suggestive of statistical heterogeneity as these tests are traditionally underpowered.

The heterogeneity between studies was also assessed using the I^2^ statistic. The I^2^ statistic is a measure of the percentage of variation in the data that is as a result of heterogeneity as opposed to chance. I^2^ values of 0-25% are considered low, 25-75% are considered moderate and over 75% are considered high heterogeneity. Possible sources of heterogeneity which we noted prior to performing our study were: 1) the method of measuring/calculating blood loss; 2) the dose of TXA and method of administration; 3) the transfusion criteria 4) patient diagnosis (osteoarthritis versus inflammatory arthritis).

In addition, two funnel-plots were constructed for the outcomes of TBL and the number of patients requiring allogeneic transfusions, in patients undergoing TKA, to assess publication bias, the tendency of studies with a negative result to not be published. The more asymmetric the funnel plot, the more potential bias.

## Results

Using our search terms, a total of 420 references were identified. Three hundred and eighty-two were excluded after applying our eligibility criteria from their titles and/or abstracts, including exclusion of duplicates. Of the remaining 38 studies, three were excluded for lack of a control group and one was not randomized. Therefore, a total of 33 studies were included. All eligible studies were English-language.

Out of the 33 studies, one was triple-blind [24], 24 were double-blind [2,4-6,8,11,12,14,17,18], [20,25-37], three were single-blind [19,38,39], and in five studies the blinding was unclear [3,40-44]. Additionally, 21 studies had a Jadad score greater than three [2,4-6,11,12,14,17-19,24-27,31], [33-38] indicating they were of high quality. Ten studies [2,3,6,18,24-26,31,35,36] used computer generated methods of randomization, one study used random number tables/lists [37] and 12 studies used sealed envelopes for allocation concealment [5,6,11,12,17,24,25,27], [29,38,40,41]. In eight studies the randomization was performed by a person/pharmacist/resident not involved in the study or the care of patients [4,5,11,17,27,29,34,35]. In eight studies the method of randomization was unclear [8,20,28,31,33,39,42,43]. Study characteristics as well as patient characteristics can be seen in Tables 1, 2, 3 and 4.

**Table 1:** Study characteristics - knee

**Study, Year**	**Patients lost to follow-up and/or excluded after randomization**	**Dose of TXA**	**Method of TXA administration**	**Control**	**Transfusion criteria**	**Method of measuring blood loss**	**Deep vein thrombosis screening method**	**DVT Prophylaxis**
**Benoni, 1996**[17]	0	10 mg/kg 12 minutes prior to tourniquet deflation + 3 hours later, extra does for severe postoperative bleeding.	IV	S	Hb < 85–100 g/L	Swabs + drain	None.	Dalteparin sodium n = 49, enoxaparin n = 37
**Hiippala, 1997**[12]	3	15 mg/kg prior to tourniquet deflation + two additional 10 mg/kg doses. 2nd dose 3–4 hours after 1st, 3rd 6–7 hours after 2nd.	IV	S	Hb < 100 g/L	Swabs + drain	Clinical exam +/− venography.	Enoxaparin
**Jansen, 1999**[2]	0	15 mg/kg 30 minutes prior to surgery and every 8 hours for 3 days	IV	S	Post-op hemocrit <26%	Swabs + drapes + drain	Clinical exam.	Fraxiparine.
**Engel, 2001**[42]		15 mg/kg + second dose of 10 mg/kg after 3 hrs.	IV	Non-TXA	Hb < 100 g/L	N/R	Clinical exam +/− venography.	N/R
**Ellis, 2001**[36]	0	15 mg/kg prior to tourniquet deflation + 10 mg/kg/hr until 12 hrs after deflation.	IV	Non-TXA	Hemocrit < 27%	N/R	N/R	Enoxaparin.
**Tanaka, 2001**[41]	0	20 mg/kg 10 minutes before surgery and/or 20 mg/kg 10 minutes prior to tourniquet release.	IV	S	N/R	Swabs + drain	Venography 7–14 days post-op + perfusion lung scan.	None
**Veien, 2002**[3]	0	10 mg/kg at end of surgery and repeated three hours later. Max dose = 1 g.	IV	S	Hemocrit < 28%	Swabs + drain	Only those with clinical signs investigated.	Fraxiparine.
**Good, 2003**[18]	4	10 mg/kg prior to tourniquet release + three hours later.	IV	S	Hb < 90 g/L	Post-op hemoglobin measurement	Clinical exam +/−ultrasound.	Fragmin
**Zohar, 2004**[35]	0	1) → 15 mg/kg prior to tourniquet release + 10 mg/kg/hr for 12 hours after release.						
			IV + O	Non-TXA	Hemocrit < 28%	Drain	Clinical exam + ultrasound 5th day post-op	Enoxaparin
		OR						
		2) → 15 mg/kg prior to tourniquet release + 10 mg/kg/hr for 2 hours after + 1 g TXA oral 6 + 12 hours after.						
		OR						
		3) → 1 g TXA orally 1 hr before surgery + after surgery every 6 hrs for 18 hrs						
**Orpen, 2006**[5]	0	15 mg/kg at time cement mixing commenced.	IV	S	Post-op Hb < 90 g/L	Swabs + drain + dressings	Ultrasound on both legs 5th day post-op.	Fragmin
**Camarasa, 2006**[31]	1	10 mg/kg prior to tourniquet deflation then + 3 hours later.	IV	S	Hb < 80 g/L or < 10 g/L dependant on symptoms.	Swabs + dressings + drain	Clinical exam +/− ultrasound.	Dalteparin sodium
**Álvarez, 2007**[6]	15	10 mg/kg + 1 mg/kg/hr perfusion.	IV	S	Hb < 80 g/L or patients showing signs/symptoms of hypoxia	Drains	Clinical exam.	Bemiparin
**Molloy, 2007**[19]	0	500 mg tranexamic acid five minutes prior to deflation of tourniquet + three hours later.	IV	Non-TXA	Post-op hemocrit < 25%	Post-op hemoglobin measuremen	Clinical exam.	Aspirin
**Kakar 2009**[4]	0	10 mg/kg prior to tourniquet deflation + 1 mg/kg/h until wound closure.	IV	S	Hb < 80 g/L or <10 g/L for those over 60 and/or those with cardiopulmonary disease.	Drains	N/R	N/R
**Wong, 2010**[11]	1	1.5 gm or 3 gm applied to joint for 5 min at end of surgery.	TP	S	Hb < 80 g/L or Hb < 100 g/L if symptoms developed.	Post-op hemoglobin measurements.	Clinical exam + ultrasound on post-op days 2 + 3.	Rivaroxaban or enoxaparin
**Sa-ngasoongsong, 2011**[24]	0	250 mg /25 mL injected after fascial closure.	IA	S	When hemocrit < 25% Hb measured, Hb < 8gm% or symptoms of anemia.	Formula	Duplex Doppler on 4th postoperative day.	Ankle motion + exercizes.
**Ishida, 2011**[43]	0	2000 mg/20 mL through intra-articular drain.	IA	S	N/R	Drains	N/R	Arteriovenous impulse system for 24 hours surgery + heparin sodium.
**Roy, 2012**[37]	0	500 mg/5 mL through two intra-articular drain.	IA	S	Hemocrit ≤28%; Drain collection ≥ 500 mL in 1st 8–10 hours + Hb loss ≥ 4 g/dL; symptoms of amenia.	Weight of swabs.	Only symptomatic cases were evaluated with ultrasonography + CT.	Stockings + early mobilization + Dalteparin

DVT – deep venous thrombosis, C – Control, TXA – ranexamic acid, S – saline, IV – intravenously, TP – topically, O – orally, N/R – not reported.

**Table 2:** Study characteristics - hip

**Study, Year**	**Patients lost to follow-up and/or excluded after randomization**	**Dose of TXA**	**Method of TXA administration**	**Control**	**Transfusion criteria**	**Method of measuring blood loss**	**Deep vein thrombosis screening method**	**DVT prophylaxis**
**Benoni, 2000**[29]	1	10 mg/kg at end of operation + again three hours later.	IV	S	Clinical condition	Swabs + drain	Clinical exam.	Klexane.
**Benoni, 2001**[33]	2	10 mg/kg - max = 1 gram over 5–10 minutes, immediately before operation started.	IV	S	Clinical decision. Usually Hb < 80–100 g/L.	Swabs + drain	Clinical exam.	Klexane.
**Husted, 2003**[25]	0	10 mg/kg for 10 minutes, 15 minutes prior to incision + 1 mg/kg/hr for 10 hours.	IV	S	Hb < 25% + clinical symptoms.	Drain + transfusions + swabs	Clinical exam +/− ultrasound + follow-up 3 months post-op.	Low molecular weight heparin.
**Yamasaki, 2004**[40]	0	1000 mg 5 minutes prior to operation.	IV	Non-TXA	N/R	Swabs + drain	Clinical exam + ascending phlebography.	None
**Garneti, 2004**[14]	0	10 mg/kg.	IV	S	N/R	Swabs + drain	Belfast scan, duplex scan or venogram - fifth postoperative day.	DTV stockings + foot pumps.
**Lemay, 2004**[34]	1	Test dose 1 mL. 10 mg/kg prior to surgery. 1 mg/kg/hr infusion until wound closure.	IV	S	90 g/L or 70 g/L – dependant on patient.	Swabs + drain	Clinical exam daily + ultrasound of both inferior limbs between post-op days 5–10 or earlier if symptoms present.	Dalteparin.
**Johansson, 2005**[26]	19	15 mg/kg single injection.	IV	S	Hb < 90 g/L	Swabs + drain + Hb balance method.	Clinical exam +/−ultrasound.	Fragmin.
**Niskanen, 2005**[30]	1	Three doses of 10 mg/kg. 1st over 5–10 minutes immediately before operation. 2nd + 3rd, 8 hrs + 16 hours after operation	IV	S	Hct 28% - 30% + clinical situation	Swabs + drain	Clinical exam +/− ultrasound.	Dalteparin.
**Claeys, 2007**[32]	0	15 mg/kg.	IV	S	Hb < 85 g/L or Hemocrit < 27%.	Swabs + drain	Clinical exam daily + ultrasound on tenth postoperative day.	Fraxiparine.
**Kazemi, 2010**[8]	0	15 mg/kg slowly 5 minutes pre-operatively	IV	S	Used allowable blood loss formula.	Swabs + drain	Clinical exam.	Enoxaparin.
**Malhotra, 2011**[20]	0	15 mg/kg 15 minutes prior to surgery.	IV	S	Reduction in Hb exceeding 25% + clinical symptoms.	Swabs + drain	Colour ultrasound on tenth post-op day.	Low molecular weight heparin + elastic leg dressing.
**McConnell, 2011**[38]	0	10 mg/kg bolus at start of surgery.	IV	Non-TXA	N/R	Change in hemocrit method.	N/R	Stockings + early mobilization + aspirin
**Imai, 2012**[39]		1 g 1) 10 minutes prior to skin closure 2) and again 2 hours later. 1 g 3) 10 minutes before surgery 4) and again 6 hours later.	IV	Non-TXA	N/R	Change in hemocrit method.	Whole body CT on 7th postoperative day.	Stockings + enoxaparin

DVT – deep venous thrombosis, C – Control, TXA – tranexamic acid, S – saline, IV – intravenously, N/R – not reported.

**Table 3:** Patient characteristics - knee

**Study, Yea**r	**Number of patients TXA/C**		**Gender distribution % male TXA /C**		**Mean age TXA,C (SD or range)**		**Mean weight (kg) - TXA, C (SD or Range)**		**TBL - TXA,C (mL) (SD)**		**P Value**	**Number of patients requiring allogeneic transfusions (TXA, C)**		**P Value**
	**TXA**	**C**	**TXA**	**X**	**TXA**	**C**	**TXA**	**X**	**TCA**	**C**		**TXA**	**C**	
**Hiippala, 1995**[27]	15	13	13	23	70 (56–82)	70 (63–78)	72 (10)	74 (10)	847 (356)	1549 (574)	<0.001	10	12	N/R
**Benoni, 1996**[17]	43	43	30	23	76 (7)	74 (7)	73 (14)	78 (16)	730 (280)	1410 (480)	<0.001	8	24	<0.001
**Hiippala, 1997**[12]	39	38	10	21	70 (7)	69 (5)	74 (13)	78 (12)	689 (289)	1509 (643)	< 0.0001	17	34	<0.0001
**Jansen, 1999**[2]	21	21	24	14	70.7 (62–80)	71.0 (64–84)	78.8 (11.0)	75.8 (12.4)	678 (352)	1419 (607)	<0.001	2	13	<0.001
**Engel, 2001**[42]	12	12	50	50	71 (9)	66 (11)	83 (12)	80 (13)	800 (295–1050)	865 (245–1370)	N/R	0	3	N/R
**Ellis, 2001**[36]	10	10	40	30	71 (5)	72 (8)	80 (12)	77 (15)	N/R	N/R	N/R	1	7	N/R
**Tanaka, 2001**[41]**	73	26	29, 32, 30		65 (59–70), , 65 (60–71), 65 (59–69)	65 (58–70)	60 (45–70), 60 (45–70), 60 (40–76)	61 (45 to 76)	720.4* (187.2)	1470 (264.8)	N/R	17, 16, 14	26	N/R
**Veien, 2002**[3]	15	15	73	93	70.5 (9.5)	69.5 (9.0)	78.7 (12.3)	74.3 (14.1)	409.7 (174.9)	761.7 (313.1)	<0.001	0	2	N/R
**Good, 2003**[18]	27	24	33	25	72 (46–83)	72 (50–84)	79 (13)	80 (13)	1045◊ (368.7 )	1426◊ (620.9 )	<0.001	3	14	<0.001
**Zohar, 2004**[35]***	60	20	30, 20, 40	35	73 (8), 69 (7), 69 (10)	73 (7)	78 (12), 75 (13), 77 (13)	75 (12)	N/R	N/R	N/R	3, 2, 4	12	N/R
**Orpen, 2006**[5]	15	14	47	79	73 (95% CI 70–78)	69 (95% CI 63–74)	83 (95% CI 69–112)	77 (95% CI 58–96)	660 (317.6)†	726 (333.0) †	0.55	1	3	N/R
**Camarasa, 2006**[31]	35	60	26	20	73 (61–84)	72 (52–85)	N/R		1095 (473)	1784 (660)	N/R	N/R		N/R
**Álvarez, 2007**[6]	46	49	15	20	71 (9)	72 (7)	N/R		1301 (621)	1744 (804)	0.002	N/R		N/R
**Molloy, 2007**[19]	50	50	N/R	N/R	N/R	N/R	N/R		1225 (499)	1415 (416)	0.041	5	11	0.79
**Kakar 2009**[4]‡	12	12	25	33	62.4 (9.4),	66.2 (4.8)	67.9 (10.8)	63.4 (7.2)	160 (87)	270 (88)	<0.05	N/R		N/R
**Wong, 2010**[11] □	64	35	19, 42 □	37	67 (11.9), 63.9 (10.6)	68.4 (10.4)	82.0 (15.6), 81.8 (14.7)	87.9 (19.2)	1295 (353.7), 1208 (376.0)	1610 (378.6)	0.0001	1, 0	2	0.083
**Ishida, 2011**[43]	50	50	12	12	73.3 (5.0)	73.5 (6.1)	59.7 (9.0)	59.7 (10.1)	N/R		N/R	0	1	N/R
**Sa-ngasoongsong, 2011**[24]	24	24	78	82	69.0 (8.2)	69.2 (7.6)	N/R	N/R	206.3 (115.4)	385.1 (145.2)	<0.0001°	1	8	0.023
**Roy, 2012**[37]	25	25	40	36	66.04 (7.15)	66.56 (8.03)	N/R	N/R	**** 109.6 ± 71.54 + 401 ± 82.44	**** 194 ± 79.66 + 870 ± 201.04	N/R	2	7	Not Significant

* - Weighted Mean Value^7^, † - Calculated from 95% CI, § Average, SD – Standard Deviation, C – Control group, TXA – Tranexamic acid group, ◊ - Median Value, DVT – deep venous thrombosis, ** (Patients received pre-op TXA, patients received intra-op TXA, patients received both pre + intra op TXA), ‡ - Unilateral TKA data reported only, □ - (Patients received 1.5 gm TXA, patients received 3 gm TXA), *** (Patients received TXA bolus + long IV, patients received TXA bolus + short IV, patients received oral TXA), N/R – not reported. **** Sum of per-operative and drain collection. ↑Patients received TXA prior to tourniquet deflation, an additional preoperative dose, an additional postoperative dose, all three doses, or locally.

**Table 4:** Patient characteristics - hip

**Study, Year**	**Number of patients TXA/C**		**Gender distribution % male TXA /C**		**Mean age TXA,C (SD or range)**		**Mean weight (kg) – TXA, C (SD or Range)**		**TBL – TXA,C (mL) (SD)**		**P Value**	**Patient requiring allogeneic transfusions (TXA, C)**		**P Value**
	**TXA**	**C**	**TXA**	**C**	**TXA**	**C**	**TXA**	**C**	**TXA**	**C**		**TXA**	**C**	
**Ekbäck, 2000**[28]	20	20	45	55	66.4 (9.0)	65.6 (8.8)	81.1 (13)	76.8 (13)	1130 (400)	1770 (523)	<0.001	5	5	N/R
**Benoni, 2000**[29]	20	19	30	58	69.5 (10)	68 (10)	76 (14),	77 (11)	990 ◊(210 ),	950 ◊(162.5)	N/R	45	79	0.05
**Benoni, 2001**[33]	18	20	50	50	66 (9.5)	68 (9.4)	79 (16)	78 (17)	759 (275.8†)	996 (398†)	0.03	22	40	0.2
**Husted, 2003**[25]	20	20	35	30	65	67	N/R		814 (1323.8†)	1231 (1692.7†)	0.001	10	35	0.04
**Yamasaki, 2004**[41]	20	20	95	90	55.5 (14.2)	61.2 (6.9)	52.6 (11.5)	54.5 (8.7)	1350 (477)	1667 (401)	<0.05	0	0	N/R
**Garneti, 2004**[14]	25	25	N/R		69.6 (11.99)	67.6 (11.4)	N/R		1443 (809)	1340 (665)	0.822	64	56	N/R
**Lemay, 2004**[34]	20	19	60	68	59.7 (10.3)	53.6 (12.8)	80.1 (15.7)	74.7 (14.1)	1308 (462)	1469 (405)	N/R	0	42	0.0012
**Johansson, 2005**[26]	47	53	53	53	69 (7)	69 (7)	81 (16)	78 (13)	969 (434)	1324 (577)	<0.001	17	43	0.009
**Niskanen, 2005**[30]	19	20	32	35	66 (9.1)	65 (8.2)	80 (19)	82 (14)	792 (390.1†)	1102 (485.2 †)	0.03	26	40	0.3
**Claeys, 2007**[32]	20	20	25	35	73 (8)	68 (11)	76 (15)	72 (16)	801 (244)	1038 (289)	0.013	5	30	<0.05
**Kazemi, 2010**[8]	32	32	72	63	46.6 (16.2)	45.4 (17.2)	72.1 (10.4),	69.9 (11.1)	1024 (544)	1399 (587)	0.024	N/R		N/R
**Malhotra, 2011**[20]	25	25	40	48	52.6 (39–72)	54.7 (40–71)	80.2 (58–100)	81.9 (62–98)	410 (300–510) – range	615 (515–750) –range	<0.5	24	72	N/R
**McConnell, 2011**[38]	22	22	32	41	N/R	N/R	N/R	N/R	930 (399†)	1200 (469†)	0.02	0	0	N/R
**Imai, 2012**[39]*	24, 20, 25, 26	22	17, 20, 16, 15	23	64.4 (49–74), 62.2 (50–85), 63.3 (47–80), 63.3 (48–81)	60.2 (54–72)	55.9 (44–86), 54.1 (40–76), 54.1 (41–70), 53.8 (44–73)	56.3 (47–80)	649, 566, 388, 418**	1026**	N/R	0	0	N/R

◊Sum of perioperative and drain medians^7^, † - Calculated from 95% CI, C – Control group, TXA – Tranexamic group, SD – Standard Deviation, DVT – deep venous thrombosis, N/R – not reported. *Patients received 1 g TXA 10 minutes prior to skin closure, 10 minutes prior to skin closure and 6 hrs later, 10 minutes prior to surgery, 10 minutes prior to surgery and 6 hrs later, respectively. ** Intra-op + post-op.

The methods of TXA administration varied between studies. TXA was administered intravenously in 29 studies [2-6,8,12,14,17-20,25-36,38-42], intra-articularly in three studies [24,37,43], orally in one study [35] and topically in one study [11].

**Figure 1:**
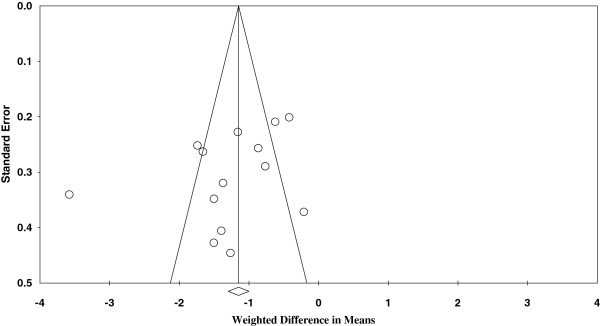
Funnel plot of standard error by weighted difference in means.

**Figure 2:**
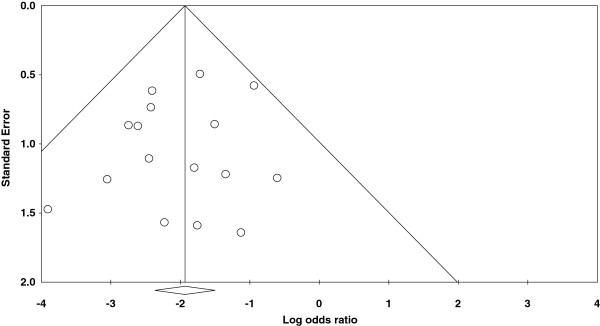
Funnel plot of standard error by log odds ratio.

Figures 1 and 2 represent funnel plots examining for potential publication bias between studies involving patients undergoing TKA. Figure 1 reports the weighted mean difference (WMD) of blood loss as a measure of the treatment effect (TXA). Figure 2 reports the logs OR of the numbers of patients requiring allogeneic transfusions as a measure of the treatment effect. Figure 1 demonstrates only minimal asymmetry and a few outliers, indicating mild publication bias. Figure 2 demonstrates moderate asymmetry, also indicating mild publication bias.

**Figure 3:**
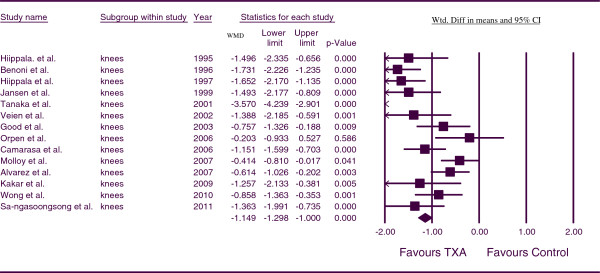
Forest plot of combined WMD values for total blood loss in knee arthroplasty.

For TBL, the combined WMD for patients undergoing TKA was found to be −1.149 (*p* < 0.001; 95% CI −1.298, -1.000) (Figure 3). This indicates that for TKA patients, blood loss was less in the TXA groups in comparison to the control group at a statistically significant level. There was a high level of statistical heterogeneity between studies (*p* = 0.000, I^2^ = 85.710). We performed a sensitivity analysis here to explore causes of heterogeneity. Pooling data from only those studies administering TXA IV and calculating TBL based on the weight change of surgical swabs and drapes as well as the drain volume, demonstrated a statistically significant benefit of TXA over control; WMD = −1.706 (*p* < .001 95% CI −1.949,-1.463).

**Figure 4:**
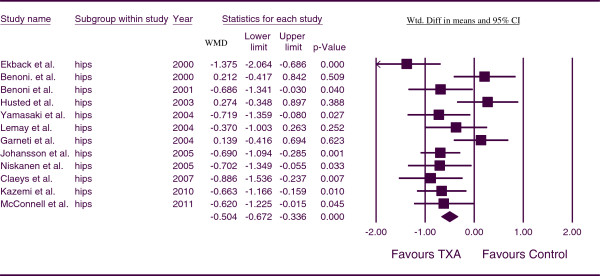
Forest plot of combined WMD values for total blood loss in hip arthroplasty.

The combined WMD value for TBL in THA was −0.504 (*p <* 0.001; 95% CI, -0.672, -0.336) (Figure 4). This indicates that, for THA patients, blood loss was less in the TXA groups in comparison to the control group at a statistically significant level. There was a moderate level of heterogeneity between studies (*p* = 0.006, I^*2*^ = 58.000).

**Figure 5:**
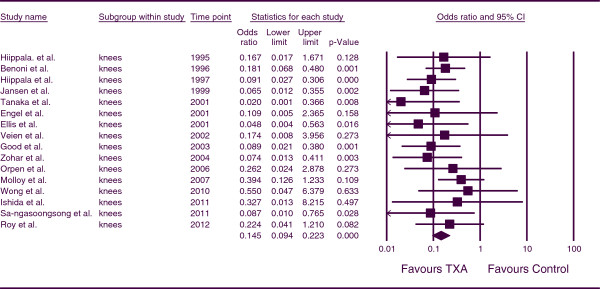
Forest plot of combined OR values for the number of patients requiring allogeneic transfusions in knee arthroplasty studies.

The combined OR for the number of patients receiving allogeneic blood transfusions for patients undergoing TKA was 0.145 (*p <* 0.001; 95% CI, 0.094, 0.223) (Figure 5). This indicates that, for TKA patients, the number of patients requiring allogeneic transfusions was less in the TXA groups in comparison to the control group at a statistically significant level. There was no heterogeneity between studies (*p* =0.801, I^*2*^ = 0.000).

**Figure 6:**
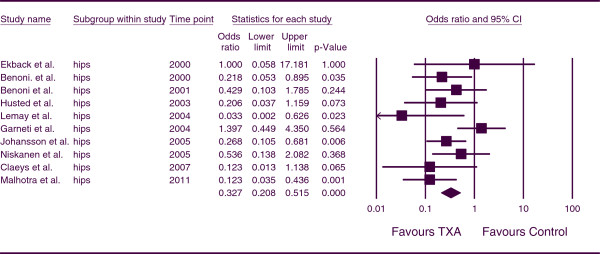
Forest plot of combined OR values for the number of patients requiring allogeneic transfusions in hip arthroplasty studies.

The combined OR for the number of patients receiving allogeneic blood transfusions for patients undergoing THA was 0.327 (*p <* 0.001; 95% CI, 0.208, 0.515) (Figure 6). This indicates that, for THA patients, the number of patients requiring allogeneic transfusions was less in the TXA groups in comparison to the control group at a statistically significant level. There was moderate heterogeneity between studies (*p* = 0.135, I^*2*^ = 34.089).

**Figure 7:**
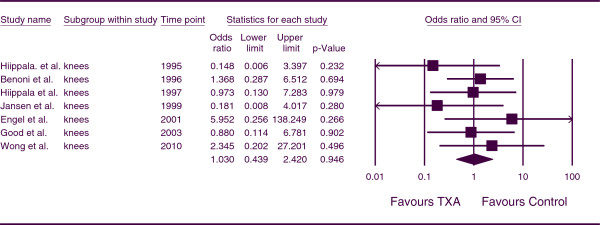
Forest plot of combined OR values for the number of patients who developed a DVT in knee arthroplasty studies.

The combined OR for the number of patients who developed a DVT for patients undergoing TKA was 1.030 (*p* = 0.946; 95% CI, 0.439, 2.420) (Figure 7). This indicates that, for TKA patients, there was no increase incidence of DVT associated with the use of TXA. There was no heterogeneity between studies (*p* =0.615, I^*2*^ = 0.000).

**Figure 8:**
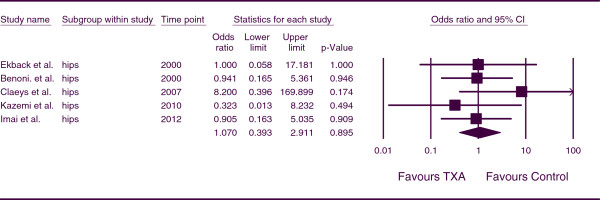
Forest plot of combined OR values for the number of patients who developed a DVT in hip arthroplasty studies.

The combined OR for the number of patients who developed a DVT for patients undergoing THA was 1.070 (*p = 0.895*; 95% CI, 0.393, 2.911) (Figure 8). This indicates that, for THA patients, there was no increase incidence of DVT associated with the use of TXA. There was no heterogeneity between studies (*p* =0.677, I^*2*^ = 0.000).

In regards to complications, overall, a total of 30 DVT, three pulmonary embolisms (PE), one myocardial infarction (patient had a history of ischaemic heart disease [27]), three wound infections, nine wound hematomas, one chest infection were reported in the TXA groups. In the control groups a total of 20 DVT, four PE, five wound infections and six wound hematomas were reported.

## Discussion

The results of our meta-analysis demonstrate a statistically significant benefit for TXA in reducing TBL and the number of patients receiving allogeneic transfusions in TKA and THA. We found the effect was even greater for TKA patients. There were also minimal differences in the incidence of thromboembolic complications with the use of TXA in our review.

In addition to TXA, other antifibrinolytic drugs, such as aprotinin, epsilon aminocaproic acid (EACA) , and fibrin spray have been used to decrease surgical blood loss [22]. Aprotinin however may cause allergic reactions [13,31], thrombosis, nephrotoxicity as well as spongiform encephalopathy [13] and has not been shown to be cost-effective [31]. Aminocaproic acid has been shown be more costly and yet less effective than TXA [13] (TXA is 7–10 times as potent [28]). TXA has also been shown to be less expensive when compared to fibrin sealants and just as effective [11,15].

In a fiscally constrained health care system, the cost benefit analysis of a therapeutic intervention is critical. Benoni et al. [17] and Good et al. [18] described total cost savings of 11, 354 SEK and £1100 respectively for patients undergoing TKA using TXA. Molloy et al. also noted an incremental cost per patient in their TXA group of only £4, compared to £380 in their fibrin spray group [19]. In their THA cohort, Benoni et al. [33] described a total savings of £625 per patient using TXA. Some have estimated a yearly savings of $65,000.00 (Can) for 1000 primary arthroplasties with routine TXA use [15]. The role of TXA use in routine primary arthroplasty appears cost effective but should be an outcome measure routinely studied in RCTs.

A single adequately powered RCT to detect a 0.5SD effect size difference in TBL (assuming 80% power and an alpha error rate of 5%) would require 63 patients per arm. By this calculation, all of the individual studies included in our meta-analysis were underpowered, suggesting that those concluding no difference between groups committed a type II beta error. This highlights the strength and importance of meta-analysis techniques as our study reviewed 33 manuscripts including a total of 1,957 patients, thereby increasing the power of our conclusion.

Additional meta-analyses and systematic reviews analyzing the efficacy of TXA in TKA and THA have been published. Zufferey et al. [44] and Kagoma et al. [22] examined the efficacy of TXA, aprontinin and EACA. However, data were pooled for orthopaedic procedures and antifibrinolytic treatment respectively. Zhang et al. [45] and Yang et al. [46] published meta-analyses analyzing blood loss, transfusion rates and rates of DVT with TXA in TKA. Data from bilateral TKAs were included, potentially leading the significant heterogeneity in mean differences of total blood loss. Cid and Lozano [47] examined transfusion rates in TKA with TXA however only included nine studies. Ho and Ismail [48] studied the effect of TXA with respect to transfusion rates. The majority of studies were for TKAs and sample size was fewer than 10 studies. Two systematic reviews and meta-analyses of TXA in TKA and THA were published in 2011 by Alshryda et al. [49] and Sukeik et al. [50] respectively. These reviews investigated the effects of TXA in TKA and THA with respect to total blood loss, post-operative blood loss, DVT and transfusion rate. Both, however, included fewer than ten studies in their analysis of total blood loss. Furthermore, Alshryda et al. included fewer than 15 studies in their analysis of allogeneic transfusions, and Sukeik et al. included fewer than 10. Despite their differences, all reviews came to conclusions analogous to our own.

There were several strengths of our review and meta-analysis. First, we performed exhaustive searches of the English and non-English languages literature to limit publication bias and pooled data from 33 manuscripts, including only RCTs. Second, many of our studies had Jadad scores of greater than three, indicating that most articles were of high quality.

### Limitations

Our paper also has limitations. We used the Jadad score for assessing the quality of the individual studies. We recognize that this tool has limitations. It has been described as simplistic as it takes into account a limited number of variables and does not take into consideration bias as a result of allocation concealment [51]. In our study, we cannot exclude the presence of additional unpublished trials that showed a negative or an equivocal difference between the intervention and control groups. We note as well some limitations related to heterogeneity. We found moderate heterogeneity between TKA studies reporting blood loss however we performed a sensitivity analysis here to explore the cause of this heterogeneity. Despite differences among the studies, our findings suggest that across the various methods of measuring TBL or routes of administration, TXA lead to decreased blood loss as compared with alternative approaches. We have presented the I^2^ value for each outcome measure analyzed. Please note we present the data despite some with high heterogeneity and suggest that the reader use caution in interpreting the results.

## Conclusion

In conclusion, we find that TXA leads to a statistically significant reduction in TBL and fewer patients requiring allogeneic transfusions, with no apparent increased risk of thromboembolic complications. Incomplete reporting of complications prevented us from pooling of this data and such larger trials are still needed to confirm the safety of TXA use in routine primary TKA and THA.

## Abbreviations

TXA: Tranexamic acid; RCT: Randomized controlled trial; TBL: Total blood loss; TKA: Total knee arthroplasty; THA: Total hip arthroplasty; DVT: Deep vein thrombosis; SD: Standard deviation; OR: Odds ratio; WMD: Weighted mean difference.

## Competing interests

There are no competing interests for any author.

## Authors’ contributions

RG conceived and designed the study, performed the statistical analysis, drafted and revised the manuscript. HE performed data acquisition, drafted the manuscript and performed the statistical analysis. SM performed data acquisition and revised the manuscript. NM participated in the study design and coordination and revised the manuscript. All authors read and approved the final manuscript.
